# Bronchocele

**Published:** 1845-01

**Authors:** 


*Bronchocele.*—Dr. B. R. Morris calls attention to rheumatism as an occasional cause of goitre, and relates three cases of the disease which he attributes to that cause. In one of these cases a cure was effected by the use of half a drachm of hydriodate of potass in an ounce of soap liniment, rubbed into the tumor every night.

*L. and E. Month. Jour. Med. Sci.*

